# Number of fragments, margin status and thermal artifacts of conized specimens from LLETZ surgery to treat cervical intraepithelial neoplasia

**DOI:** 10.1590/S1516-31802012000200004

**Published:** 2012-04-03

**Authors:** Dulcimary Dias Bittencourt, Rita Maira Zanine, Ana Martins Sebastião, Nabiha Saadi Taha, Neila Góis Speck, Julisa Chamorro Lascasas Ribalta

**Affiliations:** I Postgraduate Student Gynecological Diseases Prevention Center (Núcleo de Prevenção de Doenças Ginecológicas, Nuprev), Universidade Federal de São Paulo (Unifesp), São Paulo, Brazil.; II MD, PhD. Adjunct Professor, Department of Obstetrics and Gynecology, Universidade Federal do Paraná (UFPR), Curitiba, Paraná, Brazil.; III MD, PhD. Pathologist and Titular Professor, Centro Universidade Positivo (Unicenp), Curitiba, Paraná, Brazil.; IVMD, PhD. Assistant Professor and Head of Electrical Surgery Group, Gynecological Diseases Prevention Center (Núcleo de Prevenção de Doenças Ginecológicas, Nuprev), Universidade Federal de São Paulo (Unifesp), São Paulo, Brazil.; V MD, PhD. Affiliated Professor and Head of Lower Gynecological Tract Sector, Gynecological Diseases Prevention Center (Núcleo de Prevenção de Doenças Ginecológicas, Nuprev), Universidade Federal de São Paulo (Unifesp), São Paulo, Brazil.; VI MD, PhD. Associate Professor and Head of Gynecological Diseases Prevention Center (Núcleo de Prevenção de Doenças Ginecológicas, Nuprev), Department of Gynecology, Universidade Federal de São Paulo (Unifesp), São Paulo, Brazil.

**Keywords:** Conization, Cervical intraepithelial neoplasia, Colposcopy, Electrocoagulation, Women, Conização, Neoplasia intraepitelial cervical, Colposcopia, Eletrocoagulação, Mulheres

## Abstract

**CONTEXT AND OBJECTIVE:**

Large loop excision of the transformation zone (LLETZ) is a nontraumatic cut and coagulation method with several advantages, but it induces thermal artifacts in the cut region. The aim here was to assess the correlations of age, number of fragments, lesion grade and degree of thermal artifacts with margin quality in conized specimens from LLETZ for cervical intraepithelial neoplasia (CIN).

**DESIGN AND SETTING:**

Cross-sectional study at Universidade Federal de São Paulo (Unifesp).

**METHODS:**

The records and histopathology findings of 118 women who underwent LLETZ between 1999 and 2007 were reviewed. Age, number of fragments, lesion grade, degree of thermal artifacts and margin quality were assessed.

**RESULTS:**

The patients’ mean age was 27.14 years; 63.6% had been diagnosed with CIN II and 36.4% with CIN III. The lesion was removed as a single fragment in 79.6% of the cases. The margins were free from intraepithelial neoplasia in 85.6% and compromised in the endocervical margin in 6.8%. Fragment damage due to artifacts occurred in 2.5%. Severe artifacts occurred in 22.8%. Women aged 30 years or over presented more cases of CIN III (P < 0.0004). Neoplastic compromising of surgical margins and severe artifacts occurred more often in cases in which two or more fragments were removed, and in patients aged 30 years or over.

**CONCLUSION:**

CIN III in women aged 30 or over, when removed in two or more fragments during LLETZ, presented a greater number of compromised margins and greater severity of thermal artifacts.

## RESUMO

**CONTEXTO E OBJETIVO:**

Cirurgia de alta frequência (CAF) é um método não traumático de corte e coagulação com muitas vantagens, porém induz a artefatos térmicos na região do corte. O objetivo foi avaliar a relação entre idade, número de fragmentos, grau da lesão e grau de artefatos térmicos e a qualidade das margens das peças cirúrgicas resultantes da CAF para neoplasia intraepitelial cervical (NIC).

**TIPO DE ESTUDO E LOCAL:**

Estudo transversal na Universidade Federal de São Paulo (Unifesp).

**MÉTODOS:**

Foram revisados prontuários e laudos histopatológicos de 118 mulheres que foram submetidas a conização por cirurgia de alta frequência no período de 1999 a 2007. Idade, número de fragmentos, grau da lesão, grau de artefatos térmicos e qualidade das margens foram avaliados.

**RESULTADOS:**

A idade média das pacientes foi de 27,14 anos; 63,6% tinham diagnóstico de NIC II e 36,4% de NIC III. A lesão foi retirada com um fragmento em 79,6%. As margens estavam livres de neoplasia em 85,6% e comprometidas na margem endocervical em 6,8%. Fragmentos prejudicados por artefatos ocorreram em 2,5%. Artefatos de grau severo ocorreram em 22,8%. Mulheres com idade igual ou superior a 30 anos apresentaram mais casos de NIC III (P < 0,0004). O comprometimento neoplásico de margens cirúrgicas e artefatos de grau severo ocorreram mais vezes nos casos em que foram retirados dois ou mais fragmentos e em pacientes com idade igual ou superior a 30 anos.

**CONCLUSÃO:**

NIC III em mulheres com idade superior a 30 anos, quando retiradas em dois ou mais fragmentos na CAF, apresentaram maior número de margens comprometidas e grau severo de artefatos térmicos.

## INTRODUCTION

Electrosurgery, sometimes referred to as radiosurgery or diathermy, was initially used in 1926 by Harvey Cushing and William T. Bovi. In 1981, René Cartier proposed using small low-voltage high dielectric loops to biopsy and excise the transformation zone, requiring several passes of the loop. In 1989, Walter Prendiville used large loops, thereby removing the transformation zone as only one or two fragments. This treatment method is known as large loop excision of the transformation zone (LLETZ) or the loop electrosurgical excision procedure (LEEP).^1^^,^^2^^,^^3^^,^^4^

LLETZ is a nontraumatic cut and coagulation method with several advantages over cold knife conization, such as lower cost and shorter surgical procedure and recovery time for the patient.^1^^,^^5^ In contrast with destructive methods for lesion treatment, LLETZ enables collection of material for anatomopathological evaluation.^6^^,^^7^ However, despite all of its advantages, LLETZ induces thermal artifacts in the cut region. Their magnitude varies according to the surgeon’s ability, the number of fragments removed and the equipment used. If these fragments are not properly identified, they may cause problems for anatomopathological interpretation.^8^^,^^9^^,^^10^

## OBJECTIVE

The aim of this study was to evaluate the number of fragments, margin status and thermal artifacts of conized specimens obtained by means of LLETZ, in cases in which a single professional with experience of the technique performed the procedure.

## METHODS

We conducted a retrospective study using a convenience sample of medical records from 118 patients with high-grade cervical intraepithelial neoplasia (CIN) who underwent LLETZ between 1999 and 2007. This study was approved by the Ethics Committee of Hospital São Paulo, Universidade Federal de São Paulo (Unifesp).

The surgical procedures were performed in a private clinic by a single professional with considerable experience of the technique. The surgical method consisted of removing the transformation zone of the cervix, using a loop electrode measuring 2.0 x 0.8 cm connected to a LLETZ PLUS machine (Zinnanti Surgical Instruments, Chatsworth, California, United States).

The patients were placed in the gynecological position and the neutral plate of the machine was positioned. The cervix was visible after placement of the speculum connected to a biological vapor vacuum. 3% acetic acid was applied, and the cervical lesion was viewed under a 16 x colposcope. Lugol’s solution was applied to the cervix to mark out the cut area better. Local anesthesia was applied in the four quadrants of the cervix (12, 3, 6 and 9 o’clock) with an infiltrate of 0.5 ml of lidocaine at 2% with a vasoconstrictor. Looking through the colposcope, the surgeon activated the machine via a pedal and removed the piece using the chosen loop, which was passed across the area in a fast, continuous, firm movement, with voltage calibrated to 36 watts for cutting. Depending on the size of the lesion, it was sometimes necessary to remove more than one fragment. After excision, a 40 watt, 3 mm spherical electrode was applied to induce coagulation, followed by application of Monsel’s hyperchlorite paste to achieve hemostasis, and finally placement of a vaginal tampon. The material collected was then sent for anatomopathological analysis.

For this study, the thin sections were reviewed by two pathologists, one of them evaluated half of the sample. All 118 samples were diagnosed as severe intraepithelial neoplasia and were evaluated to determine the margin quality, degree of thermal artifacts and number of fragments. Margins considered compromised were those with cell abnormalities compatible with human papillomavirus (HPV), CIN I, CIN II or CIN III.

The degree of thermal artifacts was graded as mild, moderate or severe. Mild artifacts were characterized by a thin layer of thermal coagulation in the margin. In the mucosa, the thermal action was slight, with or without highlighting of the epithelium. This caused no problem in making the final diagnosis of the degree of compromised margin. Moderate artifacts consisted of a layer of more noticeable thermal coagulation, visible in all histological thin sections. The thermal artifacts extended and altered the shape and size of structures such as glands, cells and nuclei. Complete highlighting of the epithelium of the mucosa was observed. Making a final diagnosis was still possible. Severe artifacts consisted of the presence of extensive coagulative necrosis zones, wrinkling of the tissue, swelling and blurring of cell details. In this situation, making a final diagnosis of compromised margin was impossible. The statistical analysis used the chi-square (χ²) test. Statistical significance was accepted when P < 0.05.

## RESULTS

The patients ages were between 17 and 55 years of age, with an average of 27.1 years.

Severity of the histopathological lesion: Out of the 118 cases, 75 (63.6%) were grade 2 cervical intraepithelial neoplasia (CIN II) and the other 43 (36.4%) were grade 3 cervical intraepithelial neoplasia (CIN III).

Number of surgical fragments: In 94 cases (79.7%), a single fragment was removed, and in 24 cases (20.3%), two or more fragments were removed.

Compromising of the surgical margins: In 101 cases (85.5%), the margins were free; in eight cases (6.8%), the endocervical margins were compromised; in two cases (1.7%), the ectocervical margins were compromised; in four cases (3.4%), both margins were compromised; and in three cases (2.5%), the margins were damaged by artifacts. In none of the cases was the margin damaged by fragmentation.

Degree of thermal artifacts: In 55 cases (46.6%), the grade was mild; in 36 cases (30.5%), the grade was moderate; and in 27 cases (22.9%), the artifact grade was severe (Table 1).

Analysis of the association between age and lesion severity showed that there was a greater percentage of CIN II cases among the women less than 30 years of age. Among the women aged 30 years or over (P = 0.004), CIN III occurred in 21 cases (75.8%) (Table 2).

Among the women aged up to 30 years, excision was done as a single fragment in 82.7% and as two fragments in 17.3%. After the age of 30 years, these proportions were 73% and 27% respectively.

Analysis of the relationship between surgical margin compromising and age showed that the margins were predominantly free in the two age groups considered. However, there were more cases of compromised margins in the upper age group: 21.6% versus only 7.5%.

**Table 1.** Distribution of 118 patients according to lesion grade, number of fragments, margin quality and degree of thermal artifacts

	n	%
Histopathological grade of the cervical lesion		
Cervical intraepithelial neoplasia II	75	63.6
Cervical intraepithelial neoplasia III	43	36.4
Number of surgical fragments		
One fragment	94	79.7
Two or more fragments	24	20.3
Surgical margins		
Free	101	85.6
Endocervical compromised	8	6.8
Ectocervical compromised	2	1.7
Both compromised	4	3.4
Damaged by thermal artifacts	3	2.5
Damaged by fragmentation	0	0
Thermal artifacts		
Mild	55	46.6
Moderate	36	30.5
Severe	27	22.9

**Table 2.** Relationship between age and histopathological grade of the lesion in 118 women with cervical squamous intraepithelial neoplasia grades II and III, who underwent large loop excision of the transformation zone (LLETZ); P = 0.004 (chi-square test)

Age	Lesion grade				Total	
	Cervical intraepithelial neoplasia II		Cervical intraepithelial neoplasia III			
n	%	n	%	n	%
< 30	59	72.8	22	27.2	81	100.0
³ 30	16	43.2	21	57.8	37	100.0
Total	75	100.0	43	100.0	118	100.0

Analysis of the relationship between age and occurrence of thermal artifacts showed that 32.4% of the cases in the older women group presented severe artifacts, while this proportion was 18.5% in the younger group.

Evaluation of the relationship between the number of surgical fragments and the grade of histopathological legion showed that there was no difference in distribution between the variables. Similarly, there was no noticeable relationship between the distribution of thermal artifacts and the number of fragments.

The surgical margins were free from compromise in the majority of cases, regardless of the number of surgical fragments. However, in the group with two or more fragments, 25% of the margins were compromised, whereas in the group with only one fragment, 8.5% were compromised. In the group with two or more fragments, 4.2% of the margins were damaged by artifacts, compared with 2.1% in the group with a single fragment.

The distribution of the degree of thermal artifacts was similar in the two groups defined by surgical margin compromising, such that 100% of the margins damaged by artifacts presented severe-grade artifacts.

The distribution of CIN II and CIN III cases in relation to surgical margin compromising showed that 64.2% of the cases in the compromised margin group were CIN III, versus 35.7% with CIN II in the same group. 66.6% of the cases with margins damaged by artifacts were in the CIN III group, versus 33.3% in the CIN II group. Similar distribution of different artifact grades was registered in the two CIN groups.

## DISCUSSION

As seen in other studies, the severity of the intraepithelial lesions worsened with greater age of the women.^11^ In 79.6% of the cases, the lesion was removed as a single fragment, and this was similar to what was reported by other authors, who found that 60% to 92% of the cases resulted in single fragments.^12^^,^^13^^,^^14^ The number of fragments removed was greater in the group aged 30 years and over, and this fragmentation increased the uncertainty of the diagnosis. The success interpreted in a high number of margins was probably due to the fact that the fragments were removed in a single pass, thereby facilitating orientation. Regarding margin compromising, 11.9% of the margins were compromised by lesions, i.e. similar to values seen in other studies.^12^^,^^15^^,^^16^

In this study, the endocervical margin was the most compromised, as was seen by other authors.^17^^,^^18^^,^^19^^,^^20^ Analysis on the relationship between age and margin compromising showed a positive association, which has also been supported by other authors.^17^^,^^21^ This study presented more cases of compromised margins when the lesion grade was higher.

Despite the compromised margins in the LLETZ specimens, only a small number of patients will have residual and recurrent disease at follow-up. Thus, they do not require immediate retreatment, but their conservative follow-up should include colposcopic and cytological assessments.^22^^,^^23^

The relationship between the intensity of artifacts and patient age group showed that the older group had twice as many severe artifacts as shown by the younger group, although without statistical significance. This suggests the possibility that the cervical structure is more fibrous in older women, thereby constituting a risk factor for thermal artifacts, since such structures would retard the cutting speed. Similarly, another study showing this through evaluating the degree of thermal artifacts in different age groups was conducted by Taha et al.^24^ On the other hand, in a study by Montz et al.,^8^ artifact grade was not affected by patient age.

In this study, the severity of thermal artifacts did not present any relationship with the number of fragments. Nevertheless, many other authors have found that the thermal artifacts are milder with lower fragmentation. The quantity of artifacts might be related to the surgeon’s skill. In the cases cited here, there was no damage from fragmentation, which may be attributable to the fact that the procedure was carried out by a professional experienced in this technique, and that the loop used was large: 2.0 x 0.8 cm. Here, 2.5% of the margins were damaged by artifacts, which is similar to the proportion found by other authors.^14^^,^^25^^,^^26^

The literature presents many references to compromised margins, with percentages ranging from 0.0% to 30.5%, due to artifacts or fragmentation, thereby adversely affecting the histopathological interpretation. The highest rate (30.5%) was observed by Mathevet et al.^27^ in a study in which the equipment was not effective, and the physicians had various levels of experience with the procedure. Boardman et al.^28^ observed that 28% of their cases shown adverse effects on interpretation, with procedures conducted by physicians under training.

## CONCLUSION

We concluded that when two or more fragments are removed during LLETZ in treatment for NIC III, in women aged 30 or over, a greater number of compromised margins is observed and also greater severity of thermal artifacts in these fragments.
